# Moderate Alcohol Consumption and Risk of Depression: A Longitudinal Analysis in Community-Dwelling Older Adults

**DOI:** 10.3390/nu17162688

**Published:** 2025-08-20

**Authors:** Mohammadreza Mohebbi, Najmeh Davoodian, Shiva Ganjali, Lawrence J. Beilin, Michael Berk, Malcolm Forbes, John J. McNeil, Mark R Nelson, Joanne Ryan, Rory Wolfe, Robyn L. Woods, Mojtaba Lotfaliany

**Affiliations:** 1 Biostatistics Unit, Faculty of Health, Deakin University, Geelong, VIC 3220, Australia; m.mohebbi@deakin.edu.au; 2 Deakin University, IMPACT—The Institute for Mental and Physical Health and Clinical Translation, School of Medicine-Barwon Health Geelong, Geelong, VIC 3220, Australia; s.ganjali@deakin.edu.au (S.G.); michael.berk@deakin.edu.au (M.B.); mpforbes@deakin.edu.au (M.F.); m.lotfalianyabrandabadi@deakin.edu.au (M.L.); 3 School of Medicine, Royal Perth Hospital, University of Western Australia, Perth, WA 6000, Australia; lawrie.beilin@uwa.edu.au; 4 School of Public Health and Preventive Medicine, Monash University, Melbourne, VIC 3004, Australia; john.mcneil@monash.edu (J.J.M.); joanne.ryan@monash.edu (J.R.); rory.wolfe@monash.edu (R.W.); robyn.woods@monash.edu (R.L.W.); 5 Menzies Institute for Medical Research, University of Tasmania, Hobart, TAS 7000, Australia

**Keywords:** alcohol consumption, depression, mood disorders, causal inference, older adults

## Abstract

Background/Objectives: Evidence suggests a J-shaped association between alcohol consumption and depression, but it remains unclear whether this reflects a true causal effect, reverse causation, or methodological bias. This uncertainty is particularly relevant in older adults, who are at increased risk for both depression and alcohol-related harms. This study aimed to examine the association between varying levels of alcohol consumption and depression risk in community-dwelling older adults. Methods: We analyzed 16,563 community-dwelling older adults (mean age 75.1 ± 4.6 years) from the ASPirin in Reducing Events in the Elderly (ASPREE) trial. Alcohol intake, reported at baseline and follow-up, was categorized as abstinent, occasional, moderate, or above-guideline. Both intention-to-treat (classified by baseline alcohol consumption, regardless of later changes) and per-protocol (using annual time-updated alcohol consumption ) analyses were performed. To address confounding, informative censoring, and selection bias, we applied marginal structural models with inverse probability weighting. Results: In per-protocol analyses, abstainers (OR 1.17), occasional drinkers (OR 1.11), and above-guideline drinkers (OR 1.15) were significantly associated with a higher risk of depression compared with moderate drinkers, consistent with a J-shaped association. Sensitivity analyses excluding former drinkers and those with baseline depressive symptoms showed similar results. The association remained robust after adjusting for social isolation, social support, social interactions, physical activity, pain, sleep duration, sleep difficulties, and sleep medication use (*n* = 14,892; Australian sub-sample), and did not differ by sex. Conclusions: Moderate alcohol consumption was associated with the lowest depression risk, confirming a J-shaped relationship after comprehensive confounder adjustment.

## 1. Introduction

Depression is a primary cause of disability and functional decline worldwide [1,2]. Depression is prevalent in older adults and significantly contributes to morbidity and mortality in this group [1,3]. Therefore, enhancing the mental well-being of this demographic is crucial to prevent complications, improve late-life quality, and reduce healthcare costs [4]. The pathogenesis of late-life depression remains incompletely understood, but its etiology involves biological, psychological, and sociocultural factors [5]. Known risk factors for geriatric depression include neurobiological changes associated with aging, female sex, lower income and socioeconomic status, loneliness, lower educational attainment, smoking, chronic inflammation, and chronic disease comorbidities. Dietary exposures have also been consistently linked to variations in the risk of depressive symptoms and incident depression [6]. Among these, alcohol consumption represents a potentially modifiable risk factor [7], although its relationship with depression is complex [8]. Depressive symptoms may prompt changes in alcohol use (self-medication or, conversely, reduced intake due to illness or social withdrawal), while alcohol exposure may influence subsequent mood. Longitudinal studies in older populations underscore this complexity, reporting that depression can predict lower subsequent drinking in late life, whereas increases in alcohol intake do not uniformly precede depression onset [7,9].

Research to date on the relationship between alcohol intake and depression has been mostly undertaken in younger adult populations and has found a non-linear relationship. While compared with abstainers, light to moderate alcohol consumption may reduce depression risk, heavier consumption increases risk, forming a J-shaped pattern [8,9,10]. However, evidence specific to geriatric populations is inconsistent: some studies suggest lower depression risk among moderate drinkers, whereas others—focusing on older cohorts in Spain and the UK—find no mental-health benefit of moderate consumption compared with abstention due in part to the methodological limitations of observational designs [11]. Key methodological challenges include: (1) confounding by socioeconomic, health, and social factors; (2) misclassification of abstainers, such as the inclusion of “sick quitters”; (3) reverse causation, whereby depressive symptoms influence subsequent drinking patterns; (4) time-varying confounding, which can bias standard regression models; and (5) selection bias arising from attrition and survival effects [12,13]. These limitations leave the causal nature of the alcohol–depression association in older adults unresolved.

If moderate drinking is causally protective, mechanisms may be both physiological and sociobehavioral. Physiologically, low alcohol doses can modulate mood-related neurotransmitter systems (gamma-aminobutyric acid (GABA), glutamate, monoamines) and improve cardiometabolic profiles (e.g., HDL, insulin sensitivity, reduced inflammation), potentially lowering depression risk via vascular and inflammatory pathways. Sociobehaviorally, moderate drinking often occurs in socially engaging contexts, with higher social integration and activity levels linked to lower depression risk, whereas very infrequent or near-daily drinking is associated with greater symptom burden. Clarifying the relative contribution of these pathways is critical for interpretation and clinical guidance.

To address these limitations and considering the possible protective effect of moderate consumption on depression risk, and in light of the potential protective effect of moderate alcohol consumption on depression risk, we used large-scale longitudinal data from the ASPirin in Reducing Events in the Elderly (ASPREE) trial to investigate this association. This study aimed to determine whether moderate alcohol consumption is associated with a lower risk of depression after accounting for time-varying confounders and potential biases, thereby clarifying whether any observed protective effect reflects a potential causal relationship in older adults rather than merely biased associations driven by methodological artifacts.

## 2. Materials and Methods

### 2.1. Study Design and Participants

This study is a secondary analysis of the ASPREE trial, a double-blind, randomized, placebo-controlled study that investigated the effects of low-dose aspirin on disability-free survival, dementia, and mortality over a median duration of 4.7 years (IQR, 3.6–5.7). From March 2010 through December 2014, 19,114 community-dwelling individuals from Australia (87.4%) and the United States (12.6%), who were aged ≥70 years (≥65 for U.S. ethnic minorities), with no prior history of cardiovascular disease, dementia, or independence-limiting physical disability at trial entry, were recruited through general practitioners (Australia) and clinic-based mailing lists (United States). Participants were followed prospectively, with annual assessments, until June 2017 [14].

We also incorporated data from the ASPREE sub-study, the ASPREE Longitudinal Study of Older Persons (ALSOP), consisting of 14,892 participants. ALSOP was conducted in parallel with the main ASPREE study and exclusively included participants from Australia [15]. We included the following variables: social isolation, social support, social interactions, physical activity, experience of pain, hours of sleep, trouble falling asleep, and sleep medication from the ALSOP baseline Medical and Social questionnaires for our subgroup analysis. These variables were not included in our main models due to the smaller sample size of the ALSOP sub-study.

Participants eligible for inclusion in this study had information on alcohol consumption and depression status at baseline. Out of the 19,114 participants in the ASPREE study, four individuals were excluded due to missing information on depression status at baseline. Additionally, 2547 participants with a history of antidepressant or antipsychotic medication use were excluded to minimize confounding by indication and reverse causation. These medications are typically prescribed for depression, other mood disorders, or psychosis, and thus indicate an underlying psychiatric condition that is itself a strong predictor of future depressive episodes. Inclusion of such participants could obscure the temporal relationship between alcohol consumption and depression risk, as pre-existing mental illness and its treatment may influence both alcohol use patterns (e.g., reduction on medical advice, self-medication, or abstinence) and CES-D-10 scores, potentially leading to misclassification of exposure or outcome. Furthermore, psychotropic medications may directly alter symptom reporting, either lowering scores through treatment effects or reflecting more chronic or treatment-resistant depression. While this exclusion improves internal validity and strengthens causal inference, it may limit generalizability by removing individuals with prior psychiatric treatment who are at a higher baseline risk of depression. To address this, we accounted for antidepressant and antipsychotic use initiated during follow-up as time-varying confounders in our marginal structural models, thereby reducing potential bias from medication use arising after baseline [16]. The final analysis included 16,563 participants (Figure S1).

### 2.2. Outcome Assessment

Depression, utilizing a proxy diagnosis from a self-reported score, was the primary outcome of our study. To assess clinically relevant depressive symptoms, the Center for Epidemiologic Studies Depression Scale short version (CES-D-10) was employed. CES-D-10 is a self-report questionnaire designed to measure depressive symptoms in the general population and demonstrates robust psychometric properties, particularly in older people [17]. Participants responded to each item on the scale by rating the frequency of each mood or symptom ‘during the past week’ using a four-point scale. A total score was calculated by summing all items, with positive mood items reversed. The CES-D-10 scale ranges from 0 to 30. The CES-D-10 provides comparable accuracy (kappa = 0.75 and kappa = 0.97, *p* < 0.001 for CES-D-10 cutoffs of 8 and 10, respectively) to the full-length 20-item version of the CES-D in classifying participants with depressive symptoms. The CES-D-10 is a reliable and valid measure of depressive symptoms, with established validity and internal consistency in detecting depressive symptoms [17]. In the ASPREE study, the CES-D-10 was initially administered at baseline, and at years 1, 3, 5 and 7. Upon receipt of further funding, CES-D-10 annual assessments were added to years 2, 4 and 6. In the current study, depression risk was assessed as a binary outcome, with the scale dichotomized into two categories: non-depression (CES-D-10 scores < 8) and probable depression (CES-D-10 scores ≥ 8) based on previously suggested cutoffs. The risk of depression, including both incident and recurrent cases, was defined by the presence of clinically relevant depressive symptoms, as indicated by CES-D-10 scores of 8 or higher, assessed from baseline to annual follow-up visits. The CES-D-10 cutoff of ≥8 has been recommended in prior validation studies in older adults as a sensitive threshold for detecting probable depression, capturing both mild and moderate symptoms that are clinically relevant and associated with adverse health outcomes in late life. This threshold prioritizes sensitivity to ensure early identification of depressive symptoms in this initially healthy elderly cohort.

For our sensitivity analysis, we employed a CES-D-10 cut-off of 10, which has been widely validated as providing the optimal balance between sensitivity and specificity for identifying depressive disorders in community and older populations. A score of ≥10 closely corresponds to the standard CES-D-20 threshold of ≥16, achieving near-perfect agreement with the full version.

### 2.3. Exposure Assessment

Time-updating alcohol consumption served as the primary exposure variable in this study. We assessed alcohol intake by incorporating the frequency of weekly drinking, the average standard drinks per day, and heavy episodic drinking, defined as consuming ≥5 standard drinks on a single occasion for men or ≥4 for women, quantified in standard drinks per week. We used volume criteria consistent with the current American dietary guidelines [18]. We categorized alcohol consumption based on the previous studies as follows [8]: abstinence (no alcohol consumption), occasional consumption (average frequency of less than one day per week, with no heavy episodic drinking), moderate consumption (average frequency of at least one day per week, with average weekly drinks of ≤7 for females or ≤14 for males, and no heavy episodic drinking), and above the recommended guideline drinking (average frequency of at least one day per week, with average weekly drinks of ≥7 for females or ≥14 for males, and/or heavy episodic drinking).

The reference group was defined as moderate drinkers, in alignment with findings from a previous ASPREE study, which demonstrated a reduced risk of incident cardiovascular events and all-cause mortality associated with moderate alcohol consumption compared with never-drinkers [19]. For sensitivity analysis, we further categorized the abstinence group into never-drinkers and former alcohol consumers.

Intention-to-treat (ITT) and per-protocol (PP) analyses were used to estimate associations between alcohol consumption and depression risk. In the ITT analysis, we define exposed and non-exposed groups at baseline, regardless of whether they changed their alcohol consumption in the following waves. In PP analysis, however, annual time-updated alcohol consumption data were used to account for changes in consumption over time [20].

### 2.4. Assessment of Potential Confounders

The following variables were considered potential confounders and effect modifiers based on previous studies [8,9]: age at baseline, considered as a continuous variable; sex, categorized into male and female; education level, grouped as year 12 and under and above year 12; race/ethnicity, distinguishing White/Caucasian from others; baseline comorbidities, including type 2 diabetes, hypertension, pulmonary disease, gout, Parkinson’s disease, chronic kidney disease, dyslipidemia, metabolic syndrome, history of cancer, and gastroesophageal reflux disease; the presence of polypharmacy (use of 5 or more prescription medications); antipsychotic, antidepressant, opioids or anti-inflammatory medications use; smoking status, denoted as smoker and non-smoker; living at home with family, friends, or spouse denoted as yes and no; body mass index (BMI) was calculated by dividing weight (kg) by height squared (m^2^) and was considered a continuous variable. For our analysis, smoking status, living status, BMI, polypharmacy, antipsychotic and antidepressant medication use (participants with baseline use were excluded; initiation during follow-up was modeled as a time-varying confounder), opioid, or anti-inflammatory medications use were treated as time-updating variables, and all other potential confounders were considered time-constant, using baseline information.

### 2.5. Statistical Analysis

To estimate the associations between alcohol consumption and depression risk, we used marginal structural models (MSMs) with inverse probability weighting (IPW) to account for potential confounders. MSMs create a weighted pseudo-population in which the exposure is independent of measured confounders at each time point, allowing for unbiased estimation of causal effects when confounders are themselves affected by prior exposure.

We applied two sets of weights: (1) inverse probability of treatment weights (IPTW) to account for confounding and to balance the exposed and non-exposed groups, and (2) inverse probability of censoring weights (IPCW) to address potential selection bias due to informative censoring [21,22]. IPTWs were estimated by conducting binary logistic regression models with alcohol consumption as the dependent variable and potential predictors as independent variables. The models predicted the probability of being in each alcohol consumption group, and the IPTW was calculated as the inverse of these probabilities. IPCWs were derived from logistic regression models where the dependent variable was participation in a follow-up wave, while the independent variables were baseline covariates, as defined earlier. The model predicted the probability of being followed in each follow-up group. IPCW was calculated as the inverse of these probabilities. The final weight for each observation was calculated as the product of the IPTW and IPCW. To ensure exchangeability and avoid extreme weights, observations with weights lower than the 2nd percentile or higher than the 98th percentile were excluded. Covariate balance across alcohol consumption categories was assessed before and after employing the IPCWs using standardized mean differences (SMD); absolute SMD values < 0.10 indicated adequate balance (Figure S2) [21]. Finally, the weighted associations between alcohol consumption categories and depression risk were estimated using generalized estimating equations (GEE) models with a logit link function and binomial family, incorporating a one-year lag between alcohol exposure and depression outcome to establish temporal ordering.

### 2.6. Subgroup Analyses

We conducted the following subgroup analyses using the weighted pseudo-population: (1) We investigated the two-way interaction effect between sex and different levels of alcohol consumption; and (2) We examined potential interactions between social isolation, social support, social interactions, physical activity, experience of pain, hours of sleep, trouble falling asleep, and sleep medication using the ALSOP sub-study. Biases due to inadequate consideration of these potential confounders have long been a pitfall for epidemiologic studies of the possible effects of alcohol drinking.

### 2.7. Sensitivity Analyses

We conducted sensitivity analyses as follows: (1) All analyses were repeated after excluding former alcohol consumers (*n* = 953), who might have stopped alcohol consumption for various reasons, to mitigate potential bias from reverse causality; (2) All analyses were repeated after excluding participants with depression risk at baseline (CES-D-10 scores ≥ 8) (*n* = 1456); and (3) All analyses were repeated using a CES-D-10 score cutoff of 10. (4) We calculated the E-value to assess the robustness of our findings to potential unmeasured confounding. The E-value measures the minimum strength of association, on the odds ratio scale, that an unmeasured confounder must have with both alcohol consumption (exposure) and depression risk (outcome), conditional on the measured covariates, to explain away the observed association to the null (i.e., an OR of 1). In general, an E-value close to 1 suggests that even weak unmeasured confounding could nullify the association, whereas E-values substantially greater than the odds ratios of known, strong confounders in the field suggest greater robustness [23].

## 3. Results

### 3.1. Baseline Characteristics

Of the 16,563 participants, the mean (SD) age at study entry was 75.1 (4.6) years, 93.2% were White/Caucasian, and 53.9% were female. At baseline, approximately 26.9% of participants reported abstaining from alcohol, 21.2% reported occasional consumption, 32.6% reported moderate consumption, and 19.3% reported consumption above recommended guidelines. The pattern of alcohol consumption was consistent across follow-up waves, with the proportions of abstinent, occasional, moderate, and above-guideline drinkers displaying minimal variation. This consistency suggests stable alcohol consumption behaviors over the study period (Figure S3).

The mean CES-D-10 score was 3.0 (3.1). Compared with abstainers, occasional, and above-guideline drinkers, moderate drinkers had a higher education level, a higher proportion of males, and were more likely to live at home with family, friends, or a spouse. The number of medical comorbidities was evenly distributed across alcohol categories, with an average of 2.9 (1.6) for the total population. Overall, 75.5% had hypertension, 63.3% had dyslipidemia, and less than 20% reported each of the other chronic conditions. Notably, 28.4% of participants reported using five or more prescription medications (Table 1). Table S2 presents the characteristics of study participants after implementing Inverse Probability Weights (IPW) to each observation. Balancing confounders across different levels of alcohol consumption through implementing weights effectively nullified baseline differences between exposure groups.

A comparison of baseline characteristics between males and females showed that females had consistently higher CES-D-10 scores across all alcohol consumption categories, indicating a higher prevalence of depressive symptoms. Males had a higher prevalence of positive smoking history across all categories, with 6.7% of males consuming alcohol above the guidelines reporting a smoking history, compared to 3.5% of females (Table S1). We also presented the characteristics of the variables included in the ALSOP study in Table S3.

### 3.2. Primary Analyses

Table 2 presents the per-protocol estimates of the effect of alcohol consumption on depression risk in the unadjusted model and the IPCW-adjusted model. In the weighted sample, abstainers (adjusted OR 1.17, 95%CI 1.08–1.26), occasional drinkers (1.11, 1.03–1.19), and above-guideline drinkers (1.15, 1.07–1.25) were significantly associated with higher odds of depression than moderate drinkers. As shown in Table S5, median follow-up duration, retention, and adherence to baseline alcohol category varied across groups—highest among abstainers (89.6%) and lowest among occasional drinkers (50.2%)—highlighting potential differences in exposure stability, which were addressed in the IPW-adjusted analysis.

**Figure 1:**
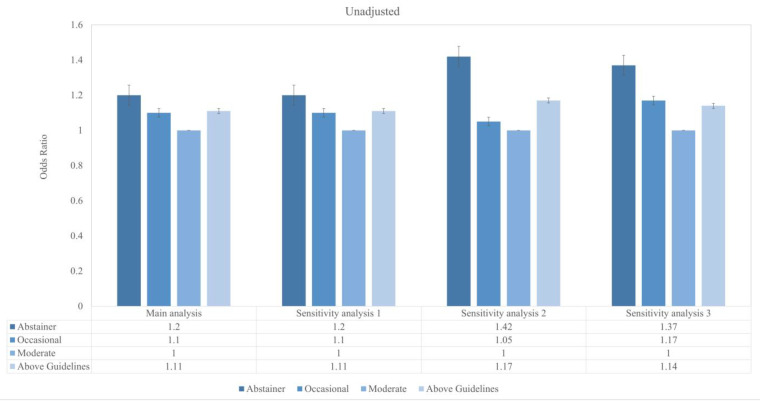
Unadjusted associations between categories of alcohol consumption and the odds of prevalent depression across main and sensitivity analyses over a median follow-up of 4.7 years. The vertical axis represents odds ratios (ORs) and 95% confidence intervals (CIs), with moderate alcohol consumption as the reference category (OR = 1.0). The horizontal axis presents results for the main analysis and three pre-specified sensitivity analyses. The main analysis included 16,563 participants. Sensitivity analysis 1 excluded former alcohol consumers (*n* = 15,610). Sensitivity analysis 2 excluded participants with CES-D-10 scores ≥ 8 at baseline (*n* = 15,107). Sensitivity analysis 3 applied an alternative CES-D-10 cutoff of ≥10 (*n* = 16,563).

**Figure 2:**
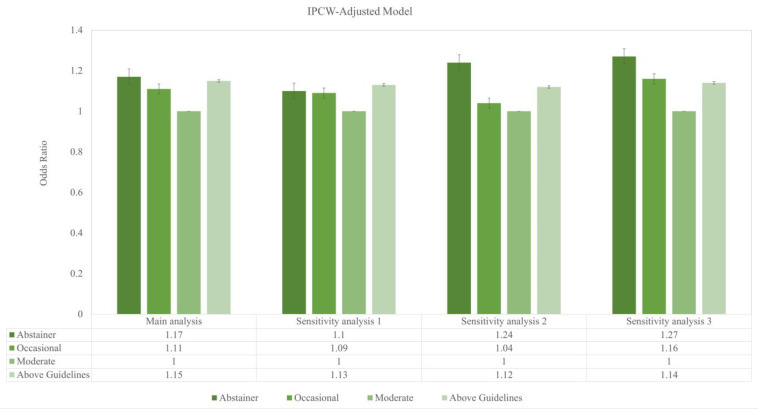
Estimated pattern of association between alcohol consumption and the risk of depression and the odds of prevalent depression across main and sensitivity analyses over a median follow-up of 4.7 years. The vertical axis represents odds ratios (ORs) and 95% confidence intervals (CIs), with moderate alcohol consumption as the reference category (OR = 1.0). The horizontal axis presents results for the main analysis and three pre-specified sensitivity analyses.

Figure 1 reveals a J-shaped association between alcohol consumption categories and risk of depression, illustrating that moderate drinkers were associated with the lowest depression rate, followed by occasional drinkers. Conversely, individuals consuming alcohol above the recommended guidelines and those abstaining from alcohol were associated with the highest rates of depression. This J-shaped pattern persists in the weighted sample, as evidenced in Figure 2.

The intention-to-treat analysis in each alcohol consumption category is presented in Table S7. Compared with the moderate alcohol consumption group, abstainers (1.18, 0.98–1.41), occasional drinkers (1.26, 1.03–1.54), and above-guideline drinkers (1.43, 1.16–1.71) were associated with increased risk of depression over a 4.7-year period.

The effect modifications of social isolation, social support, social interactions, physical activity, experience of pain, hours of sleep, trouble falling asleep, and sleep medication use were not statistically significant (Table S4). Furthermore, we explored the two-way interaction between sex and alcohol consumption categories. The analysis indicated that the interaction was not statistically significant for abstainers (0.92, 0.76–1.12), occasional drinkers (0.95, 0.80–1.13), and above-guideline drinkers (0.86, 0.71–1.04). A post hoc power calculation indicated that the study had 80% power to detect a 16% change in OR, showing sufficient statistical power to detect a small effect size in sex-specific effects. These results suggest that the relationship between depression and alcohol consumption was not significantly different in female and male populations.

Table S5 presents unadjusted descriptive characteristics relevant to the PP analysis, including median follow-up duration, unadjusted depression rates, retention rates, and the proportion of participants maintaining their baseline alcohol consumption category. Adherence to baseline category was highest among abstainers (89.6%) and lowest among occasional drinkers (50.2%), while retention ranged from 71.0% to 92.6% across categories. These descriptive data provide context for the PP analysis and allow evaluation of potential bias related to differential follow-up, attrition, or adherence.

### 3.3. Sensitivity Analyses

Similar results were obtained for the analysis after excluding former drinkers (sensitivity analysis 1), with moderate drinkers estimated to have significantly lower odds of depression than abstainers (1.10, 1.01–1.21), as well as above-guideline drinkers having higher odds (1.13, 1.03–1.23) (Table 2).

The results of sensitivity analyses 2, which excluded participants with depressive symptoms at baseline (CES-D-10 scores ≥ 8), also showed that moderate drinkers were significantly associated with a reduced odds of depression, comparable to those of abstainers (1.24, 1.11–1.38), occasional drinkers (1.04, 0.93–1.17), and above-guideline drinkers (1.12, 0.99–1.26) (Table 2).

The results of sensitivity analysis 3, using a CES-D-10 cutoff of 10, were consistent with the main analysis. Moderate drinkers were significantly associated with a lower odd of depression, comparable to those of abstainers (1.27, 1.13–1.42), occasional drinkers (1.16, 1.04–1.31), and above-guideline drinkers (1.14, 1.01–1.29) (Table 2).

E-values analysis suggests that the odds ratios for depression risk are relatively robust to the effect of unknown or unmeasured confounders. Specifically, E-values were moderately high for all comparisons in the main analysis, indicating robustness of the effects. E-values must be interpreted within the context of the present study. In our IPCW-adjusted model, the E-values were 1.61 for abstainers, 1.46 for occasional drinkers, and 1.51 for above-guideline consumers. Their magnitude should be considered in relation to the associations of other known risk factors for depression. When most other risk factors have odds ratios around 1.1–1.2, E-values in the range of 1.4–1.5 are considered relatively large because unmeasured confounding must have substantially greater effects than most known risk factors to explain away the reported associations.

An E-value of 1.51 for the above guideline drinkers, for instance, shows that to fully account for the estimated odds ratio, one or more unmeasured confounders would need to almost double the chance of an individual belonging to the above guideline drinking group and the chance of experiencing depression. In addition, E-values ranged from 1.2 to 2.2 across all models, indicating that any unmeasured confounder would need to have a strong correlation with both exposure and outcome to shift the odds ratio point estimate to 1.0 (Table 2).

## 4. Discussion

This study applied a causal inference analysis, using intention-to-treat and per-protocol approaches with inverse probability weighting, to examine the association between alcohol consumption and depression risk among community-dwelling older adults in Australia and the United States. We performed both intention-to-treat and per-protocol analyses, with the per-protocol as the primary outcome. The per-protocol analysis that considers change over time in alcohol consumption while dealing with missing data and drop-outs by IPCW is likely a better estimation of the real-world effects of alcohol. The results from both analyses demonstrate that moderate alcohol consumption is associated with a lower risk of depression compared to both abstainers and those who consume above-recommended levels. This association remained stable after excluding the former drinkers or participants with depression at baseline. Our findings provide preliminary evidence to suggest that the observed relationships may reflect a possible causal link between alcohol consumption and the risk of depression in older adults, rather than being merely artifacts driven by methodological biases.

Consistent with prior research findings [8], both abstainers and above-guideline drinkers were more likely to experience depression. Previous studies have demonstrated that alcohol abstinence is more prevalent among depressed older adults compared to their non-depressed counterparts. While heavy drinking does not significantly differ between depressed and non-depressed groups, it is associated with more severe depression symptoms. Moreover, having an alcohol use disorder at least doubles the odds of depression [24]. These associations are concordant with the known neurophysiological and metabolic alterations caused by alcohol exposure [7].

The pathophysiological relationship between alcohol consumption and depression involves a complex interplay of neurotransmitter disruptions, inflammatory responses, stress response alterations, and neuroimmune interactions [25]. Alcohol modulates crucial neurotransmitters such as GABA and dopamine, which play a role in mood regulation. While moderate alcohol intake may enhance these neurotransmitter systems and offer protective effects, excessive and chronic consumption is associated with significant disruptions [25].

Moreover, heavy drinking initiates an immune response, releasing pro-inflammatory cytokines that induce neuroinflammation, contributing to depressive symptoms [11,24]. This inflammatory state is exacerbated by alterations in the hypothalamic–pituitary–adrenal (HPA) axis, which regulates stress responses, and the activity of neuroimmune mediators, further complicating alcohol’s impact on brain function. The release of circulating cytokines and other neuroimmune mediators from excessive alcohol consumption may lead to severe depressive symptoms. While heavy alcohol consumption has been associated with an adverse immune response [26], moderate alcohol consumption has been shown to lower inflammatory markers, including interleukin-6 and C-reactive protein (CRP) [27]. Furthermore, these biological interactions underscore the intricate link between varying levels of alcohol consumption and risk of depression [28].

Another possible pathway for the putative benefits of moderate alcohol drinking is that it facilitates social interaction, an established protective factor against depression [29]. We adjusted our model for social factors, which have often complicated epidemiological studies exploring the potential effects of alcohol consumption. However, in the current study, factors such as social isolation, social support, and social interactions were not significant effect modifiers of the alcohol-depression relationship. Possible explanations for the non-significant role of social factors in the alcohol-depression relationship include: (1) the benefits of moderate drinking on mood and stress reduction might operate primarily through biochemical pathways, such as the enhancement of neurotransmitter function and inflammatory response modulation, which could overshadow the subtler effects of social interaction; (2) the measurement of social variables might not have captured the quality and meaningfulness of social interactions, which can vary widely and have different impacts on mental health; and (3) the complex interplay of individual behaviors and broader societal norms that govern alcohol use [30]. Cultural and social norms, which vary widely between communities, shape the expectations and acceptability of drinking behaviors, potentially influencing the mental health outcomes associated with alcohol use. These norms can sometimes overshadow the direct effects of social interactions related to drinking [31]. These suggest that while social factors are crucial in general mental health research, their influence might be more complex in the context of alcohol-related studies.

In our study, neither social and cultural factors nor physical activity demonstrated independent associations with depression risk, suggesting that their role in late-life depression may be less pronounced or not fully captured by our measurement approach.

Physical activity showed no modifying effects on the alcohol-depression relationship. Exercise is established as an effective treatment for depression in older adults [32]. However, there are fewer studies examining its effects on individuals with alcohol use disorders [33,34]. Exercise exerts its beneficial impact on well-being on multiple levels. It can enhance self-esteem, improve physical self-perception, and contribute to a sense of purpose [35]. This may be mediated by increases in brain-derived neurotrophic factor (BDNF)—a protein that plays a crucial role in neuronal survival, development and plasticity [36]. Several factors may explain why physical activity did not modify the alcohol-depression relationship in this study. Firstly, physical activity was measured relying on self-reported and non-validated questionnaires, which are susceptible to systematic over-reporting due to recall error, social desirability bias, and misclassification of activity intensity. Secondly, the type and intensity of physical activity may not have reached the moderate-to-vigorous levels necessary to significantly influence depressive symptoms. Finally, individual responses to physical activity may vary [37,38].

The effect of pain in the alcohol-depression relationship was not significant. This is in keeping with previous research in younger adults that found the relationship between alcohol misuse and depressive symptoms was independent of chronic pain [39]. We also found no significant mediating effect of sleep quality on the alcohol–depression relationship. While moderate alcohol intake may facilitate shorter sleep onset latency, excessive consumption is often associated with poor sleep quality, which can contribute to depression [40]. This absence of significance could be due to variability in individual sleep patterns or differences in the measurement of sleep quality, which may not have captured the full impact of alcohol consumption on sleep disturbances and their subsequent influence on depression [41].

The influence of sex on the alcohol–depression relationship remains inconsistent. Some studies report stronger associations in women, others in men [42,43]. Biological sex differences in alcohol pharmacokinetics—such as lower total body water, reduced gastric alcohol dehydrogenase activity, and higher peak blood alcohol concentrations in women—may increase susceptibility to alcohol’s neurotoxic and pro-inflammatory effects, whereas men, particularly in late life, tend to consume alcohol more frequently and above guideline levels, increasing cumulative exposure [10]. Sex differences in depression vulnerability further complicate interpretation, with women exhibiting higher lifetime prevalence and men often presenting with under-recognized or atypical symptoms. These opposing patterns, combined with our use of sex-specific alcohol thresholds, may attenuate measurable interaction effects. Although we observed no statistically significant sex differences, potential residual confounding means this finding should be interpreted cautiously.

This study has the following strengths and limitations. A key strength is the application of a causal inference framework, incorporating IPW and longitudinal modeling, to mitigate confounding, selection bias, and differential outcome ascertainment. Importantly, the IPW process for addressing confounding bias assumes no unmeasured confounders, and this assumption’s sensitivity was evaluated by calculating E-values. Additional strengths include a large, well-characterized, community-based sample of older adults, rigorous baseline screening, minimal missing information, and detailed information on modifying factors, including sleep, and physical and social factors. Moreover, this study provides evidence of the relationship between alcohol consumption and the development of depression in older adults, who often experience higher levels of comorbidity and cognitive impairment.

However, this study has some major limitations. The selection procedure of this clinical trial may introduce a bias toward healthy volunteers, potentially limiting generalizability. The CES-D-10, while validated as a screening tool, does not provide a formal depression diagnosis, possibly leading to misclassification. Additionally, reliance on self-reported data for alcohol consumption may result in under-reporting, potentially due to the social stigma associated with heavy drinking. Potential lifestyle confounders such as physical activity may be under-reported and others unrecognized. Additionally, since alcohol metabolism varies by genetics and the study primarily included individuals of European descent, the findings may not be generalizable to Asian or African populations.

The proportion of alcohol abstainers increased over time, primarily reflecting a decline in occasional drinkers transitioning to abstinence (Figure S3). This shift may reflect survival bias, whereby individuals who live longer are more likely to stop alcohol consumption in later years, or a change in drinking habits of older adults due to health concerns, medical advice, or altered social circumstances. This potential bias should be considered when interpreting the observed J-shaped pattern between alcohol consumption and depression.

## 5. Conclusions

In conclusion, this study demonstrated a J-shaped association between alcohol consumption and depressive symptoms in a large cohort of initially healthy older adults, with moderate consumption associated with the lowest risk. The findings suggest that the protective effects of moderate consumption may not be solely attributable to methodological bias. E-value analysis indicates that residual confounding is likely minimal; however, results should still be interpreted with caution given the observational design and reliance on self-reported exposures. These findings also emphasize the importance of monitoring above-guideline drinking, particularly in psychiatric and geriatric care.

## Figures and Tables

**Table 1:** Baseline characteristics of the study population. Number (%) or Mean (SD).

Characteristics		Abstainer (*n* = 3756)	Occasional (*n* = 4195)	Moderate (*n* = 5340)	Above Guidelines (*n* = 3272)	Total (*n* = 16,563)
Age Mean (SD)		75.3 (5.0)	75.2 (4.6)	75.0 (4.4)	75.0 (4.3)	75.1 (4.6)
Education level	Above year 12	1377 (36.7%)	1779 (42.4%)	2589 (48.5%)	1482 (45.3%)	7227 (43.6%)
Race/ethnicity	White/Caucasian	3093 (84.3%)	3811 (92.1%)	5155 (96.8%)	3215 (98.5%)	15,274 (93.2%)
	Other	574 (15.7%)	328 (7.9%)	168 (3.2%)	48 (1.5%)	1118 (6.8%)
Sex	Female	2508 (66.8%)	2616 (62.4%)	1775 (33.2%)	2033 (62.1%)	8932 (53.9%)
Body mass index	Mean (SD)	28.7 (5.3)	28.5 (4.9)	27.6 (4.1)	27.2 (4.4)	28.0 (4.7)
Positive history of smoking		135 (3.6%)	145 (3.5%)	173 (3.2%)	154 (4.7%)	607 (3.7%)
Living at home with family, friends, or spouse	Yes	2410 (64.2%)	2651 (63.2%)	3902 (73.1%)	2301 (68.9%)	11,264 (68.0%)
Hypertension	Yes	2870 (76.4%)	3110 (74.1%)	3897 (73.0%)	2465 (75.3%)	12,342 (74.5%)
Diabetes	Yes	553 (14.7%)	449 (10.7%)	500 (9.4%)	223 (6.8%)	1725 (10.4%)
Pulmonary disease	Yes	516 (13.7%)	566 (13.5%)	738 (13.8%)	484 (14.8%)	2304 (13.9%)
Chronic kidney disease	Yes	504 (13.4%)	477 (11.4%)	708 (13.3%)	212 (6.5%)	1901 (11.5%)
History of cancer ^a^	Yes	670 (18.0%)	737 (17.6%)	1108 (20.8%)	640 (19.6%)	3155 (19.1%)
Parkinson’s Disease	Yes	37 (1.0%)	39 (0.9%)	50 (0.9%)	24 (0.7%)	150 (0.9%)
Gout	Yes	168 (4.5%)	200 (4.8%)	409 (7.7%)	273 (8.3%)	1050 (6.3%)
Dyslipidemia	Yes	2291 (61.3%)	3586 (62.1%)	3224 (60.9%)	2311 (71.2%)	10,412 (63.3%)
Gastro-esophageal reflux disease	Yes	1040 (27.7%)	1128 (26.9%)	1391 (26.0%)	875 (26.7%)	4434 (26.8%)
Metabolic syndrome	Yes	1480 (49.9%)	1596 (38.9%)	1715 (32.8%)	1008 (31.4%)	5789 (35.7%)
Number of comorbidities	Mean (SD)	3.0 (1.7)	2.9 (1.6)	2.8 (1.6)	2.9 (1.5)	2.9 (1.6)
Polypharmacy	Yes	1249 (33.3%)	1249 (29.8%)	1296 (24.3%)	903 (27.6%)	4697 (28.4%)
Opioids	Yes	114 (3.0%)	129 (3.1%)	112 (2.1%)	73 (2.2%)	428 (2.6%)
Anti-inflammatory	Yes	572 (15.2%)	641 (15.3%)	898 (16.8%)	674 (20.6%)	2785 (16.8%)
CES-D-10	Mean (SD)	3.1 (3.2)	3.0 (3.2)	2.8 (2.9)	3.1 (3.1)	3.0 (3.1)

The body mass index was calculated as weight in kilograms divided by height in meters squared. Hypertension is defined as systolic blood pressure ≥140 mmHg or diastolic blood pressure ≥90 mmHg or on treatment for high blood pressure. Diabetes mellitus is defined as self-report of diabetes or fasting glucose ≥126 mg/dL or on treatment for diabetes. Chronic kidney disease is defined as an estimated glomerular filtration rate <60 mL/min/1.73 m^2^ or urinary albumin to creatinine ratio ≥3 mg/mmol. Dyslipidemia is defined as cholesterol-lowering medications or serum cholesterol ≥212 mg/dL (≥5.5 mmol/L; Australia) and ≥240 mg/dL (≥6.2 mmol/L; U.S.) or low-density lipoprotein > 160 mg/dL (>4.1 mmol/L). Polypharmacy is defined as taking ≥5 prescription medications daily. ^a^ Defined as the diagnosis of any cancer during the study period or a history of a cancer diagnosis. Notes: CES-D-10 = Center for Epidemiologic Studies Short Depression Scale.

**Table 2:** The association between alcohol consumption and risk of depression.

	Alcohol Categories	Unadjusted				IPCW-Adjusted Model			
		Odds Ratio	95% CI	E-value	*p*-value	Odds Ratio	95% CI	E-value	*p*-value
Main analysis									
	Abstainer	1.20	1.10–1.30	1.69	<0.001	1.17	1.08–1.26	1.61	<0.001
	Occasional	1.10	1.02–1.19	1.43		1.11	1.03–1.19	1.46	
	Moderate	Reference				Reference			
	Above Guidelines	1.11	1.02–1.20	1.46		1.15	1.07–1.25	1.51	
Sensitivity analysis 1									
	Abstainer	1.20	1.10–1.30	1.69	<0.001	1.10	1.01–1.21	1.43	0.002
	Occasional	1.10	1.02–1.19	1.43		1.09	1.00–1.18	1.40	
	Moderate	Reference				Reference			
	Above Guidelines	1.11	1.02–1.20	1.46		1.13	1.03–1.23	1.51	
Sensitivity analysis 2									
	Abstainer	1.42	1.28–1.57	2.19		1.24	1.11–1.38	1.78	
	Occasional	1.05	0.94–1.17	1.28		1.04	0.93–1.17	1.24	
	Moderate	Reference			0.002	Reference			0.002
	Above Guidelines	1.17	1.05–1.31	1.61		1.12	0.99–1.26	1.49	
Sensitivity analysis 3									
	Abstainer	1.37	1.23–152	2.08	<0.001	1.27	1.13–1.42	1.85	0.002
	Occasional	1.17	1.06–1.30	1.61		1.16	1.04–1.31	1.59	
	Moderate	Reference				Reference			
	Above Guidelines	1.14	1.02–1.27	1.53		1.14	1.01–1.29	1.53	

Sensitivity analysis 1 involved excluding participants with a history of alcohol consumption. Sensitivity analysis 2 involved excluding participants with depression at baseline (CES-D-10 scores ≥ 8). Sensitivity analysis 3 utilized a CES-D-10 score cutoff of 10 for additional robustness. Inverse probability of censoring weights (IPCW).

## Data Availability

The individual participant data that underlie the results reported in this article will be made available after deidentification. Requests for data access will be via the ASPREE Principal Investigators, with details for applications provided through the website, www.ASPREE.org (accessed on 1 October 2023), and in accordance with the NIH policy on data sharing: details available at https://grants.nih.gov/grants/policy/data_sharing/ (accessed on 1 October 2023). Data availability will commence on publication of this article. These data will be available upon request to investigators whose proposed use of the data, registered as a project through the ASPREE Access Management Site: https://ams.aspree.org/public/ (accessed on 1 October 2023), has been approved by a review committee. These data will be available through a web-based data portal safe haven, based at Monash University, Australia.

## Supplementary Materials

Attached. The following supporting information can be downloaded at: https://www.mdpi.com/article/10.3390/nu17162688/s1. Table S1: Baseline characteristics of the study population stratified by sex. Number (%) or Mean (SD); Table S2: Baseline characteristics of the study population after assigning the inverse probability weights. Number (%) or Mean (SD); Table S3: Baseline distribution of social support, social interaction, pain, self-reported physical activity, sleep duration, and quality; Table S4: Assessing effect modification of social support, social interaction, pain, self-reported physical activity and sleep duration, and quality and alcohol consumption on depression outcome; Table S5: Unadjusted follow-up distribution and outcome, retention, and intervention adherence rate at the end of follow-up; Table S6: Distribution of weights; Table S7: The effect of baseline alcohol consumption level on the risk of depression; Figure S1: Flow diagram of study participants included in this analysis; Figure S2: Standardized mean differences for baseline characteristics before and after adjustment. Values less than 0.1 after adjustment (between the dashed reference lines) indicate good balance; Figure S3: Pattern of alcohol consumption at baseline and across the follow-up waves.
