# Supplementary material for: Moderate Alcohol Consumption and Risk of Depression: A Longitudinal Analysis in Community-Dwelling Older Adults
Source: Nutrients. 2025 Aug 20;17(16):2688. doi: 10.3390/nu17162688 (PMC12389151; doi:10.3390/nu17162688)
Supplement: Supplementary file 1 [file nutrients-17-02688-s001.zip › nutrients-3802032-supplementary.pdf]

Supplementary appendix

Moderate Alcohol Consumption and Risk of Depression: A Longitudinal Analysis in Community-Dwelling Older Adults

Contents list of supplementary material: CLICK on any item to jump to it.

Supplementary Tables

Table S1 Baseline characteristics of the study population stratified by sex. Number (%) or Mean (SD). 3
Table S2 Baseline characteristics of the study population after assigning the inverse probability weights. Number (%) or Mean (SD). 5
Table S3 Baseline distribution of social support, social interaction, pain, self-reported physical activity, sleep duration, and quality. 7
Table S4 Assessing effect modification of social support, social interaction, pain, self-reported physical activity and sleep duration, and quality and alcohol consumption on depression outcome 8
Table S5 Unadjusted follow-up distribution and outcome, retention, and intervention adherence rate at the end of follow-up. 12
Table S6 Distribution of weights 13
Table S7 The effect of baseline alcohol consumption level on the risk of depression. 14

Supplementary Figures

Figure S1 Flow diagram of study participants included in this analysis. 2
Figure S2 Standardized mean differences for baseline characteristics before and after adjustment. Values less than 0.1 after adjustment (between the dashed reference lines) indicate good balance. 10
Figure S3 Pattern of alcohol consumption at baseline and across the follow-up waves. 11

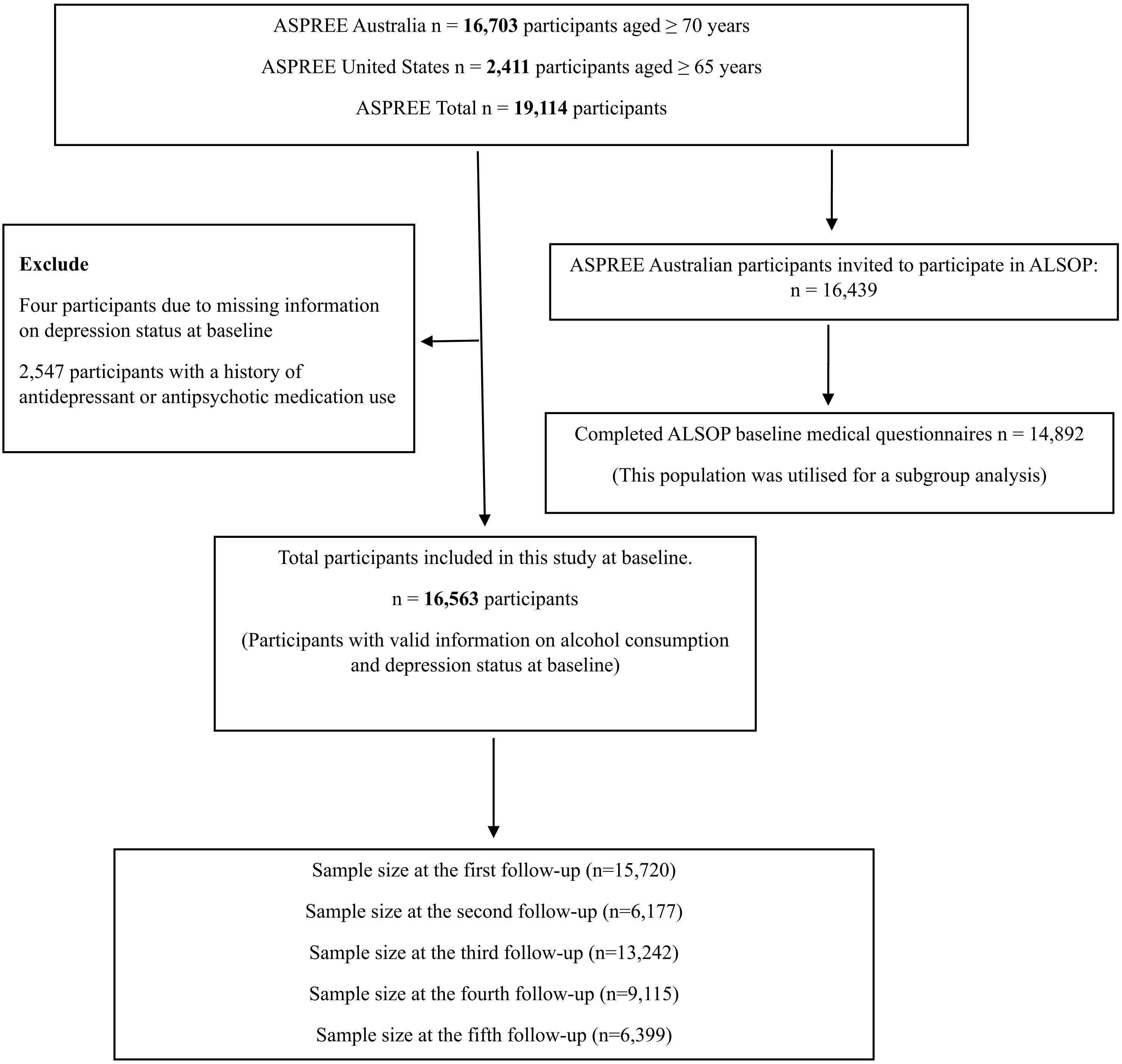

Figure S1 Flow diagram of study participants included in this analysis.

Table S1 Baseline characteristics of the study population stratified by sex. Number (%) or Mean (SD).

**Characteristics**	**Male**				**Female**			
	**Abstainer**	**Occasional**	**Moderate**	**Above guidelines**	**Abstainer**	**Occasional**	**Moderate**	**Above guidelines**
**Number of participants**	1248	1579	3565	1239	2508	2616	1775	2033
**Age (y) (mean ± SD)**	75.2 (4.8)	75.1 (4.5)	75.0 (4.4)	74.4 (4.0)	75.4 (5.0)	75.2 (4.7)	74.9 (4.3)	75.4 (4.5)
**Education level (Above year 12)**	491 (39.3%)	672 (42.6%)	1785 (50.1%)	503 (40.6%)	886 (35.3%)	1107 (42.3%)	804 (45.3%)	979 (48.2%)
**Race**								
**White/ Caucasian**	1029 (84.1%)	1442 (93.0%)	3448 (97.0%)	1213 (98.5%)	2064 (84.5%)	2369 (91.5%)	1707 (96.4%)	2002 (98.6%)
**Non-White**	194 (15.9%)	109 (7.0%)	105 (3.0%)	19 (1.5%)	380 (15.5%)	219 (8.5%)	63 (3.6%)	29 (1.4%)
**Body mass index**								
**Mean (SD)**	28.1 (4.4)	28.1 (4)	27.3 (3.7)	28.1 (3.8)	29.0 (5.6)	28.8 (5.3)	27.5 (4.7)	26.6 (4.5)
**Positive history of smoking (n, %)**	72 (5.8%)	67 (4.2%)	125 (3.5%)	83 (6.7%)	63 (2.5%)	78 (3.0%)	48 (2.7%)	71 (3.5%)
**Living at home with family, friends, or spouse (n, %)**	951 (76.2%)	1225 (77.6%)	2870 (80.5%)	994 (80.2%)	1459 (58.2%)	1426 (54.5%)	1032 (58.1%)	1307 (64.3%)
**Hypertension**	941 (75.4%)	1162 (73.6%)	2642 (74.1%)	995 (80.3%)	1929 (76.9%)	1948 (74.5%)	1255 (70.7%)	1470 (72.3%)
**Diabetes Mellitus**	187 (15.0%)	214 (13.6%)	401 (11.2%)	132 (10.7%)	366 (14.6%)	235 (9.0%)	99 (5.6%)	91 (4.5%)
**Pulmonary disease**	159 (12.7%)	194 (12.3%)	455 (12.8%)	205 (16.5%)	357 (14.2%)	372 (14.2%)	283 (15.9%)	279 (13.7%)
**Chronic kidney disease**	337 (27.0%)	356 (22.5%)	645 (18.1%)	156 (12.6%)	167 (6.7%)	121 (4.6%)	63 (3.5%)	56 (2.8%)
**History of cancer**	254 (20.5%)	315 (20.0%)	785 (22.1%)	277 (22.4%)	416 (16.7%)	422 (16.2%)	323 (18.2%)	363 (17.9%)
**Parkinson's Disease**	14 (1.1%)	18 (1.1%)	35 (1.0%)	9 (0.7%)	23 (0.9%)	21 (0.8%)	15 (0.8%)	15 (0.7%)
**Gout**	108 (8.7%)	141 (8.9%)	378 (10.6%)	218 (17.6%)	60 (2.4%)	59 (2.3%)	31 (1.7%)	55 (2.7%)
**Dyslipidaemia**	571 (46.1%)	812 (51.8%)	1920 (54.3%)	786 (63.8%)	1720 (68.8%)	1774 (68.4%)	1304 (74.0%)	1525 (75.7%)

**Gastro-oesophageal reflux disease**	307 (24.6%)	386 (24.4%)	913 (25.6%)	328 (26.5%)	733 (29.2%)	742 (28.4%)	478 (26.9%)	547 (26.9%)
**Metabolic syndrome**	480 (39.2%)	617 (39.9%)	1209 (34.7%)	473 (38.8%)	990 (40.3%)	979 (38.2%)	506 (29.2%)	535 (26.9%)
**Number of comorbidities**								
**Mean (SD)**	3.0 (1.7)	2.9 (1.7)	2.9 (1.7)	3.2 (1.6)	3.1 (1.6)	2.9 (1.6)	2.7 (1.6)	2.7 (1.5)
**Polypharmacy**	319 (25.6%)	361 (22.9%)	775 (21.7%)	341 (27.5%)	930 (37.1%)	888 (33.9%)	521 (29.4%)	562 (27.6%)
**Opioids**	29 (2.3%)	33 (2.1%)	63 (1.8%)	26 (2.1%)	85 (3.4%)	96 (3.7%)	49 (2.8%)	47 (2.3%)
**Antiinflammatory**	176 (14.1%)	213 (13.5%)	590 (16.5%)	275 (22.2%)	396 (15.8%)	428 (16.4%)	308 (17.4%)	399 (19.6%)
**CES-D-10**								
**Mean (SD)**	2.6 (3)	2.6 (2.9)	2.6 (2.9)	2.6 (2.8)	3.3 (3.3)	3.2 (3.3)	3.0 (2.9)	3.3 (3.2)

Table S2 Baseline characteristics of the study population after assigning the inverse probability weights. Number (%) or Mean (SD).

Characteristics	Abstainer (N=4,363)	Occasional (N=4,715)	Moderate (N=5,845)	Above guidelines (N=4,023)	Total (N=18,946)
**Age at randomisation (years)**					
**Mean (SD)**	75.2 (4.6)	75.0 (4.6)	75.1 (4.5)	75.6 (4.8)	75.2 (4.6)
**Education level**					
**Above year 12**	1514 (41.3%)	1833 (44.7%)	2440 (46.8%)	1352 (39.7%)	7138 (43.6%)
**Race**					
**White/ Caucasian**	3312 (92.3%)	3707 (91.6%)	4891 (94.1)	3255 (95.8%)	15165 (93.4%)
**Other**	277 (7.7%)	338 (8.4%)	306 (5.9%)	142 (4.2%)	1063 (6.6%)
**Sex**					
**Female**	2202 (60.1%)	2469 (60.2%)	1859 (35.6%)	2334 (68.5%)	8865 (54.1%)
**Body mass index**					
**Mean (SD)**	27.9 (4.9)	28.4 (4.9)	27.8 (4.2)	27.8 (4.7)	28.0 (4.6)
**Smoking history**					
**positive history of smoking**	151 (4.1%)	150 (3.6%)	174 (3.3%)	138 (4.0%)	613 (3.7%)
**Living at home with family, friends, or spouse**					
**Yes**	2487 (67.8%)	2631 (64.2%)	3723 (71.4%)	2254 (66.1%)	11095 (67.7%)
**Hypertension**					
**Yes**	2748 (74.9%)	3035 (74.0%)	3822 (73.3%)	2577 (75.6%)	12182 (74.3%)
**Diabetes Mellitus**					
**Yes**	387 (10.6%)	428 (10.4%)	563 (10.8%)	304 (8.9%)	1682 (10.3%)
**Pulmonary disease**					
**Yes**	512 (14.0%)	556 (13.6%)	716 (13.7%)	479 (14.1%)	2263 (13.8%)
**Chronic kidney disease**					
**Yes**	395 (10.8%)	461 (11.2%)	767 (14.7%)	279 (8.2%)	1902 (11.6%)
**History of cancer**					
**Yes**	699 (19.2%)	732 (17.9%)	1056 (20.3%)	630 (18.5%)	3116 (19.1%)
**Parkinson**					
**Yes**	31 (0.8%)	37 (0.9%)	48 (0.9%)	28 (0.8%)	144 (0.9%)
**Gout**					
**Yes**	216 (5.9%)	219 (5.3%)	370 (7.1%)	205 (6.0%)	1009 (6.2%)
**Dyslipidaemia**					
**Yes**	2342 (64.2%)	2555 (62.8%)	3073 (59.4%)	2252 (66.6%)	10222 (62.8%)
**Gastro-oesophageal reflux disease**					
**Yes**	996 (27.2%)	1085 (26.5%)	1369 (26.3%)	923 (27.1%)	4373 (26.7%)
**Metabolic syndrome**					
**Yes**	1251 (34.8%)	1523 (38.0%)	1751 (34.3%)	1167 (34.9%)	5692 (35.5%)
**Number of comorbidities**					
**Mean (SD)**	2.9 (1.6)	2.9 (1.6)	2.9 (1.6)	2.9 (1.6)	2.9 (1.6)
**Polypharmacy**					
**Yes**	1066 (29.1%)	1190 (29.0%)	1342 (25.7%)	1015 (39.8%)	4613 (28.1%)
**Opioids**					
**Yes**	88 (2.4%)	121 (2.9%)	126 (2.4%)	103 (3.0%)	437 (2.7%)

**Antiinflammatory**					
**Yes**	623 (17.0%)	647 (15.8%)	843 (16.2%)	614 (18.0%)	2728 (16.6%)
**CES-D-10**					
**Mean (SD)**	2.9 (3.1)	2.9 (3.2)	2.8 (2.9)	3.2 (3.1)	2.9 (3.1)

Body mass index was calculated as weight in kilograms divided by height in metres squared.

Hypertension is defined as systolic blood pressure ≥ 140 mmHg or diastolic blood pressure ≥ 90 mmHg or on treatment for high blood pressure.

Diabetes mellitus is defined as self-report of diabetes or fasting glucose ≥ 126 mg/dL or on treatment for diabetes.

Chronic kidney disease is defined as an estimated glomerular filtration rate <60 ml/min/1.73 m2 or urinary albumin to creatinine ratio ≥3 mg/mmol.

Dyslipidaemia is defined as cholesterol-lowering medications or serum cholesterol ≥212 mg/dL (≥5.5 mmol/L; Australia) and ≥ 240 mg/dL (≥6.2 mmol/L; U.S.) or low-density lipoprotein >160 mg/dL (>4.1 mmol/L).

Polypharmacy is defined as taking≥5 prescription medications daily.

a Defined as the diagnosis of any cancer during the study period or a history of a cancer diagnosis.

Notes: CES-D-10 = Center for Epidemiologic Studies Short Depression Scale.

Table S3 Baseline distribution of social support, social interaction, pain, self-reported physical activity, sleep duration, and quality.

**Characteristics**		**Total (*n* = 14,892)**
Social Isolation [a]		
	Yes	11128
Social support [a]		
	Yes	11070
Social interactions [a]		
	None	227
	One	252
	Two	883
	Three-Four	3206
	Five-Eight	3732
	Nine or more	4479
Experience pain [b]		
	Yes	6452
Physical activity [c]		
	I rarely did any physical activity	186
	I did no more than light physical activity	4110
	I did no more than moderate physical activity	6396
	I did regular vigorous physical activity	1993
Hours of sleep [d]		
	Less than 4 hours	127
	4-6 hours	3086
	6-8 hours	8746
	8-10 hours	2540
	10-12 hours	89
Trouble falling asleep [d]		
	Never	2623
	Rarely (Less than once a month)	5338
	Sometimes (1-3 times a month)	3922
	Often (Once a week or more)	1851
	Always (Most nights)	815
Sleep medication use [d]		
	Never	11042
	Rarely (Less than once a month)	1592
	Sometimes (1-3 times a month)	863
	Often (Once a week or more)	436
	Always (Most nights)	664

[a] Social health - From the validated Revised Lubben Social Network Scale (LSNS) (1). Social isolation was defined as engaging in community activities less than once per month and having contact with four or fewer relatives and close friends in a month. Social support was defined as having four or more relatives or close friends with whom private matters could be discussed, in combination with friends or relatives who could be called upon for help. Social interactions were defined as the number of friends contacted at least once a month.

[b] Self-reported data regarding pain experienced on most days were collected from the 14-page baseline ALSOP Medical Health Questionnaire. The question we included in our study was: 'Do you experience pain on most days?' Response options were 'Yes' or 'No.' Respondents were asked to complete the subsequent questions if they answered 'Yes' (2).

[c] Physical activity was assessed through self-classification of usual level of activity (3).

[d] Quality of sleep was assessed through set of 10 questions modified from the Pittsburgh Sleep Quality Index (4), including sleep duration and frequency of sleep problems (3).

Table S4 Assessing effect modification of social support, social interaction, pain, self-reported physical activity and sleep duration, and quality and alcohol consumption on depression outcome

		Abstainer			Occasional			Above Guideline		
		**Odds Ratio**	**95% CI**	**p-value**	**Odds Ratio**	**95% CI**	**p-value**	**Odds Ratio**	**95% CI**	**p-value**
**Social isolation**										
	Yes	0.98	0.65-1.48	0.934	0.88	0.60-1.30	0.527	1.41	0.98-2.02	0.062
**Social support**										
	Yes	0.96	0.77-1.19	0.694	0.85	0.69-1.04	0.111	0.91	0.74-1.12	0.371
**Social interactions**										
	None	Reference								
	One	1.54	0.60-3.93	0.367	2.34	0.93-5.85	0.070	2.48	0.97-6.34	0.057
	Two	1.63	0.76-3.50	0.210	1.89	0.91-3.91	0.088	1.48	0.70-3.14	0.308
	Three-Four	1.53	0.75-3.13	0.240	1.63	0.83-3.18	0.154	1.46	0.72-2.97	0.291
	Five-Eight	1.38	0.67-2.83	0.580	1.43	0.73-2.79	0.300	1.14	057-2.31	0.710
	Nine or more	1.59	0.78-3.24	0.204	1.40	0.71-2.75	0.329	1.13	0.56-2.30	0.728
**Experience pain**										
	Yes	1.14	0.92-1.14	0.218	1.08	0.88-1.31	0.467	1.12	0.92-1.38	0.266
**Hours of sleep**										
	Less than 4 hours	Reference								
	4-6 hours	0.56	0.24-1.32	0.183	0.55	0.25-1.21	0.136	0.84	0.38-1.89	0.676
	6-8 hours	0.50	0.22-1.18	0.113	0.55	0.25-1.20	0.113	0.75	0.34-1.67	0.482
	8-10 hours	0.61	0.25-1.49	0.283	0.57	0.25-1.30	0.181	0.88	0.38-2.04	0.760
	10-12 hours	1.52	0.15-15.64	0.727	2.88	0.34-24.72	0.335	3.12	0.32-29.96	0.325
**Physical activity**										
	I rarely did any physical activity	Reference								
	I did no more than light physical activity	1.20	0.64-2.25	0.578	1.16	0.61-2.21	0.657	0.72	0.40-1.29	0.271
	I did no more than moderate physical activity	1.19	0.63-2.23	0.597	1.05	0.55-1.99	0.891	0.71	0.40-1.26	0.243
	I did regular vigorous physical activity	1.27	0.64-2.52	0.501	1.21	0.61-2.39	0.583	0.73	0.38-1.37	0.325

**Trouble falling asleep**										
	Never	Reference								
	Rarely	0.96	0.68-1.36	0.839	1.08	0.78-1.51	0.635	0.97	0.68-1.39	0.871
	Sometimes	1.07	0.76-1.51	0.701	1.19	0.86-1.67	0.294	0.94	0.66-1.34	0.741
	Often	0.86	0.59-1.25	0.433	1.08	0.76-1.54	0.679	0.96	0.66-1.39	0.836
	Always	1.10	0.72-1.69	0.661	1.26	0.84-1.89	0.257	0.87	0.55-1.38	0.556
**Sleep medication use**										
	Never	Reference								
	Rarely	1.12	0.83-1.51	0.453	0.99	0.76-1.30	0.956	0.99	0.76-1.30	0.967
	Sometimes	0.84	0.58-1.21	0.349	0.83	0.58-1.18	0.297	1.02	0.73-1.41	0.918
	Often	1.12	0.67-1.86	0.674	0.90	0.61-1.34	0.607	0.96	0.66-1.39	0.814
	Always	0.89	0.57-1.38	0.597	0.85	0.53-1.36	0.496	0.71	0.44-1.12	0.142

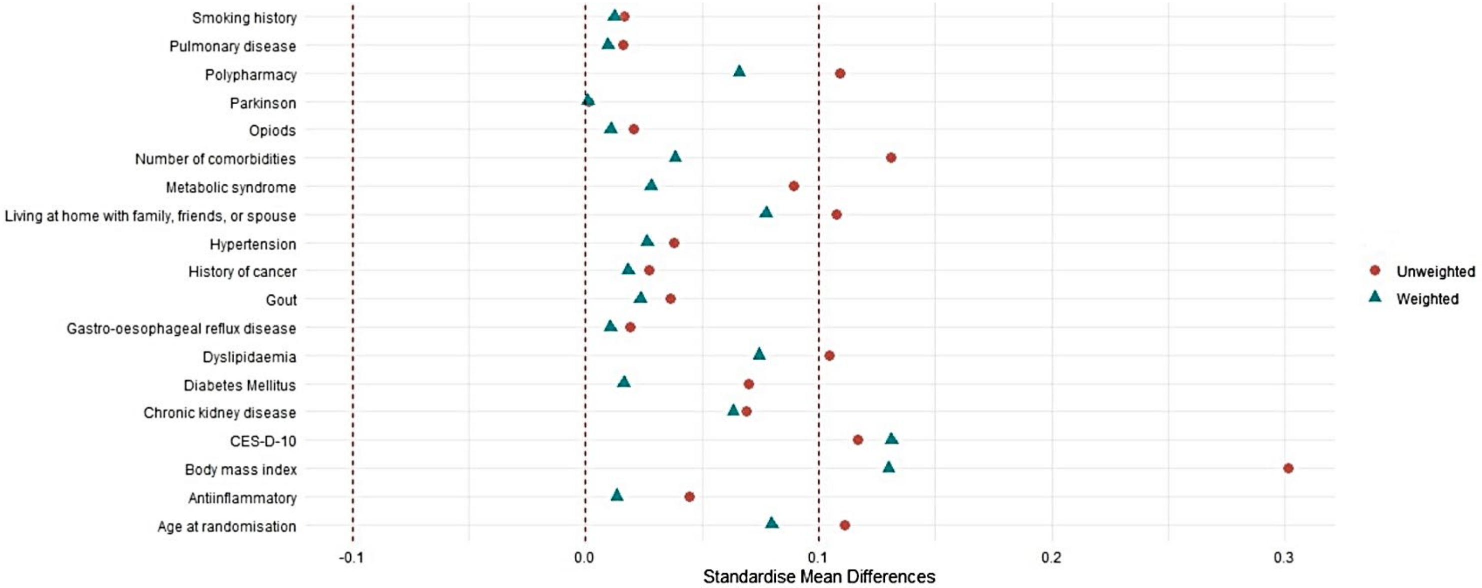

Figure S2 Standardized mean differences for baseline characteristics before and after adjustment. Values less than 0.1 after adjustment (between the dashed reference lines) indicate good balance.

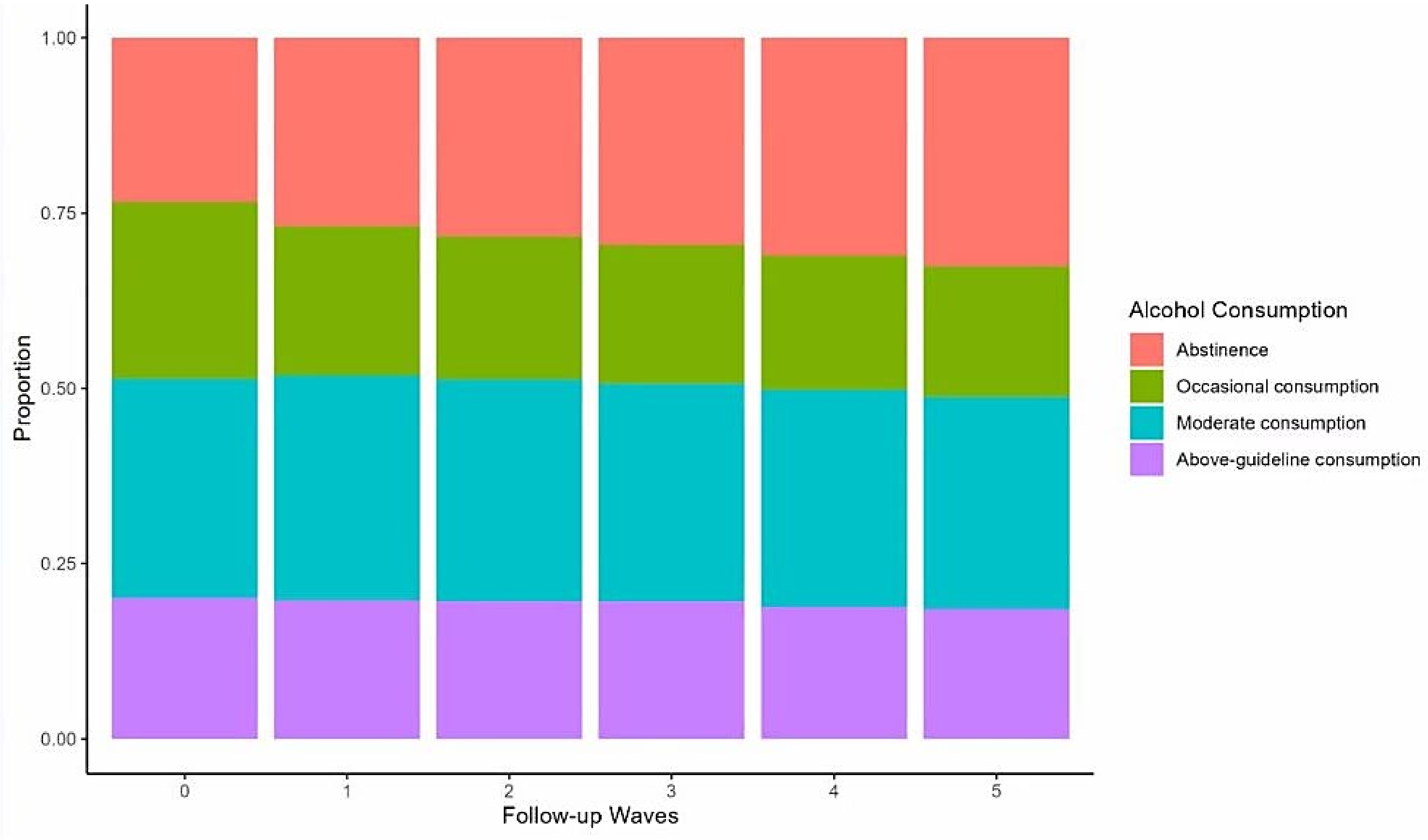

Figure S3 Pattern of alcohol consumption at baseline and across the follow-up waves.

Table S5 Unadjusted follow-up distribution and outcome, retention, and intervention adherence rate at the end of follow-up.

**Treatment initiation (Alcohol consumption categorization at baseline)**	**Unadjusted medians follow-up in years (IQR)**	**Unadjusted depression rate**	**Unadjusted retention rate**	**Unadjusted rate of maintained use of the assigned level of alcohol**
**Abstinence**	4.05<br>(2.94, 5.30)	14.8%	92.6%	89.6%
**Occasional consumption**	4.11<br>(2.95, 5.41)	12.4%	71%	50.2%
**Moderate consumption**	4.11<br>(2.97, 5,42)	11.2%	75.3%	71.2%
**Above-guideline consumption**	4<br>(2.92-5.25)	13.5%	89.7%	71.6%

Table S6 Distribution of weights

	Mean (STD)	Median (IQR)	5th percentile	95th percentile	Minimum	Maximum
**Weightings for baseline characteristics**						
**Overlap weights**	0.99 (0.32)	0.93 (0.82, 1.58)	0.60	1.53	0.30	8.33
**Weightings for treatment adherence during follow-up**						
**Unstabilised inverse probability of treatment adherence weights**	1.74 (0.89)	1.18 (0.7, 1.57)	0.72	3.06	0.31	36.50
**Stabilised inverse probability of treatment adherence weights**	1.47 (0.66)	1.16 (0.8-1.5)	1.00	2.88	1.00	8.85

Table S7 The effect of baseline alcohol consumption level on the risk of depression.

Alcohol categories	Odds Ratio	95% CI	E-value*	P
Abstainer	1.18	0.98-1.41	1.64	0.081
Occasional	1.26	1.03-1.54	1.83	0.028
Moderate	Reference			
Above Guidelines	1.43	1.16-1.77	2.21	0.001

* Larger E-values indicate it is less likely that unmeasured confounding entirely explains the association.

References

1. Joyce J, Ryan J, Owen A, Hu J, McHugh Power J, Shah R, et al. Social isolation, social support, and loneliness and their relationship with cognitive health and dementia. International journal of geriatric psychiatry. 2022;37(1).
2. Tse AWW, Ward S, McNeil JJ, Barker A, Cicuttini F, Fitzgibbon BM, et al. Severe low back or lower limb pain is associated with recurrent falls among older Australians. Eur J Pain. 2022;26(9):1923-37.
3. McNeil JJ, Woods RL, Ward SA, Britt CJ, Lockery JE, Beilin LJ, et al. Cohort profile: The ASPREE longitudinal study of older persons (ALSOP). International journal of epidemiology. 2019;48(4):1048-9h.
4. Buysse DJ, Reynolds III CF, Monk TH, Hoch CC, Yeager AL, Kupfer DJ. Quantification of subjective sleep quality in healthy elderly men and women using the Pittsburgh Sleep Quality Index (PSQI). Sleep. 1991;14(4):331-8.
